# Methodological Flaws, Conflicts of Interest, and Scientific Fallacies: Implications for the Evaluation of Antidepressants’ Efficacy and Harm

**DOI:** 10.3389/fpsyt.2017.00275

**Published:** 2017-12-07

**Authors:** Michael P. Hengartner

**Affiliations:** ^1^Department of Applied Psychology, Zurich University of Applied Sciences (ZHAW), Zurich, Switzerland

**Keywords:** antidepressants, selective serotonin reuptake inhibitor, efficacy, harms, suicide, serious adverse events, bias

## Abstract

**Background:**

In current psychiatric practice, antidepressants are widely and with ever-increasing frequency prescribed to patients. However, several scientific biases obfuscate estimates of antidepressants’ efficacy and harm, and these are barely recognized in treatment guidelines. The aim of this mini-review is to critically evaluate the efficacy and harm of antidepressants for acute and maintenance treatment with respect to systematic biases related to industry funding and trial methodology.

**Methods:**

Narrative review based on a comprehensive search of the literature.

**Results:**

It is shown that the pooled efficacy of antidepressants is weak and below the threshold of a minimally clinically important change once publication and reporting biases are considered. Moreover, the small mean difference in symptom reductions relative to placebo is possibly attributable to observer effects in unblinded assessors and patient expectancies. With respect to trial dropout rates, a hard outcome not subjected to observer bias, no difference was observed between antidepressants and placebo. The discontinuation trials on the efficacy of antidepressants in maintenance therapy are systematically flawed, because in these studies, spontaneous remitters are excluded, whereas half of all patients who remitted on antidepressants are abruptly switched to placebo. This can cause a severe withdrawal syndrome that is easily misdiagnosed as a relapse when assessed on subjective symptom rating scales. In accordance, the findings of naturalistic long-term studies suggest that maintenance therapy has no clear benefit, and non-drug users do not show increased recurrence rates. Moreover, a growing body of evidence from hundreds of randomized controlled trials suggests that antidepressants cause suicidality, but this risk is underestimated because data from industry-funded trials are systematically flawed. Unselected, population-wide observational studies indicate that depressive patients who use antidepressants are at an increased risk of suicide and that they have a higher rate of all-cause mortality than matched controls.

**Conclusion:**

The strong reliance on industry-funded research results in an uncritical approval of antidepressants. Due to several flaws such as publication and reporting bias, unblinding of outcome assessors, concealment and recoding of serious adverse events, the efficacy of antidepressants is systematically overestimated, and harm is systematically underestimated. Therefore, I conclude that antidepressants are largely ineffective and potentially harmful.

Treatment guidelines for major depression (MD) in Europe and the United States typically recommend antidepressant medication with or without psychotherapy (1). In particular, the American Psychiatric Association (APA) advises antidepressants as first-line treatment for all forms of MD, including mild episodes (2). The APA further recommends antidepressants not only for acute treatment but also for continuation therapy (approximately 4–9 months) and maintenance therapy (several years up to indefinite time). Overall, the APA treatment guidelines for adult MD provide are very favorable risk–benefit analysis for selective serotonin reuptake inhibitors (SSRIs) and serotonin norepinephrine reuptake inhibitors (SNRIs), suggesting that these drug classes are highly effective, well tolerated, and safe. However, paradoxically the massive increase in antidepressant prescription rates over the last three decades did not translate into measurable public health benefits (3–5). From a public mental health perspective, we would expect that effective antidepressants reduce the prevalence and burden of MD, unless the scientific evidence is unreliable. In the following, I will therefore provide a critical re-examination of antidepressants’ efficacy and safety. I will mostly rely on evidence from randomized controlled trials (RCTs), as these are considered gold-standard to establish causality and efficacy of clinical interventions. Although RCTs are less prone to bias than observational studies, they also have limitations, in particular in industry-funded pharmacological research (6–8). A special emphasis will hence be given to the unduly ties between scientific psychiatry and the pharmaceutical industry (9–11). I will first examine antidepressants’ efficacy for acute treatment, proceed with a review of their efficacy for continuation and maintenance therapy, and close with an outline of severe harms. Scientific biases will be discussed in context, as they substantially inflate antidepressants’ public health significance.

## Acute Therapy

Research funded by the pharmaceutical industry is systematically biased toward their marketed products (12–15). That is, the estimated efficacy of pharmaceutical products is significantly higher when the research was funded by the industry compared to non-industry funding, but this difference is not attributable to differences in the study quality (13, 14). For instance, research funded and conducted by the NIMH largely failed to demonstrate a clear difference between antidepressants and placebo, despite adequate sample sizes and strong RCT methodology [e.g., Ref. (16, 17)]. Research conducted by authors with financial conflicts of interest (COI) related to the pharmaceutical industry is likewise systematically biased in favor of the industry’s vested interests (18–20). That is, efficacy of pharmacological treatments is overestimated, whereas harms are underreported. Due to the pervasive entanglement of psychiatry with the pharmaceutical industry (9–11, 21), these biases undermine the validity of the scientific literature on antidepressants’ efficacy. For instance, it is now clearly established that many industry-funded antidepressant trials were never published, and if published, some results were inadequately presented in a favorable way (15, 22, 23). That is, trials with negative results are either not published or negative results are distorted to appear positive (7, 24). Outcome reporting bias is a common scientific flaw and means that authors conceal the effect of the prespecified primary efficacy outcome and instead choose to report the most convenient from different secondary outcomes (25). Also, harms and serious adverse events of antidepressants are often not reported, and such concealment is strongly related to authors’ financial COI and industry funding (26). Another common flaw is to report efficacy based on drug-placebo differences in response and remission rates (27). To come at binary constructs such as response and remission, continuous symptom rating scales are dichotomized along arbitrary cut-offs. However, methodologists have vigorously advised against the use of dichotomization (28–30) because it produces, among others, systematically inflated effect sizes (31–33). Most short-term efficacy RCTs were conducted to receive marketing approval from regulatory agencies. To ensure that drug-placebo differences in the outcome are clearly attributable to the intervention, efficacy RCT use preselected groups of participants tested under ideal clinical settings (34). Therefore, trial conditions markedly deviate from real-world clinical settings, and the included participants are not representative of the patient population seen in routine clinical practice. Specifically, RCT exclude a majority of MD patients due to comorbid disorders and suicidal ideation (35). Included participants are less impaired and have a higher level of functioning (36, 37), and it has been demonstrated that they respond better to antidepressants than the average real-world patient (36).

Meta-analyses that include unpublished trials and that examine mean differences in continuous depression rating scales report statistically significant but marginally small differences between antidepressants and placebo (23, 38–40). More recent studies have suggested that drug-placebo differences are larger when instead of heterogeneous sum-scores, which also include somatic symptoms, only depressive core symptoms were considered (41, 42). However, the results barely support this conclusion, because estimated effect sizes are still small and considered clinically insignificant per convention. But what exactly means clinically significant? In general, clinical significance refers to an effect size *d* or *g* > 0.5 or a difference >3 points on the Hamilton Depression Scale for Depression (HAMD) (38). However, empirical evidence has suggested that at least 7 points on the HAMD or an effect size >0.87 is necessary for a clinician to observe a minimal improvement in depression symptoms (43). Based on these criteria, the efficacy of antidepressants is impossible to discern from placebo effects in any meta-analysis conducted thus far.

Some authors deem the small difference between antidepressants and placebo a methodological artifact (27, 44–46). For instance, Chen et al. (47) re-analyzed a double-blind RCT with the treatment arms antidepressant (sertraline), St John’s Wort, and placebo, with respect to patients’ treatment beliefs. Depression symptom reduction did not differ between treatment arms. However, independent of actual treatment received, patients who believed they receive placebo showed less improvement, and patients who correctly guessed that they receive placebo improved even less. One hypothesis states that the assumption of blinding is violated even in double-blind RCT, because both patients and outcome assessors may correctly guess who receives placebo due to a suspicious lack of side effects (44). Such unblinding effects inflate the apparent efficacy of antidepressants, because unblinded outcome assessors systematically overestimate the efficacy of experimental interventions (48, 49). Taking into account the unblinding bias, Gotzsche (50) calculated that the average efficacy of antidepressants does not differ from placebo. More reliable than differences in subjective rating scales are objective outcomes such as premature treatment termination. Several meta-analyses did not detect a noticeable difference between antidepressant and placebo arms with respect to overall dropout rates in short-term RCT (51–53). Assuming that patients prematurely terminate a free treatment only if they perceived it as useless or even harmful (54), these findings indicate that the average patient experiences no clear benefits from antidepressants.

## Continuation and Maintenance Therapy

There are two trial designs to examine effects of long-term antidepressant use. The first is the long-term parallel-arm efficacy trial, where responders to both antidepressants and placebo are followed up. The second, much more common design, is the discontinuation trial, where antidepressant responders are randomly assigned to either continued therapy or rapid discontinuation and switching to placebo. In a recent meta-analysis of long-term parallel-arm RCT of 6–8 months duration, no difference between groups was found with respect to both remission and treatment discontinuation rates (55). Meta-analyses of discontinuation trials suggest that long-term antidepressant use may prevent relapse, but only very few trials have empirically examined that effect for 2 years or more (56, 57). In addition, effects in discontinuation trials are difficult to interpret, because they exclusively include participants who remitted during acute open-label antidepressant treatment. Therefore, it is not known how many patients would have relapsed if they had remitted spontaneously, i.e., without prior acute antidepressant therapy (55). This is a serious issue, because it has been argued that antidepressants prospectively increase the relapse rate due to pharmacodynamics (58, 59). Another problem with discontinuation trials is the rapid discontinuation of antidepressants and immediate switching to placebo given the ambiguous nature of subsequently emerging symptoms (60). Research has shown that antidepressants may cause severe withdrawal symptoms after discontinuation (61, 62), which in some cases persist for months and therefore are easily misdiagnosed as depression relapse (63). That is, a substantial portion of depression relapses in discontinuation trials are in fact discontinuation or withdrawal syndromes. In support of this notion, it has been shown that rapid discontinuation of antidepressants, relative to gradual tapering, prospectively increases the risk of depression reoccurrence (64), but note that even very slow tapering may cause severe mood disturbances (61, 63). It has further been shown that the preventive effects of antidepressants dissipate after 1–3 months (65). At the latest after 6 months, the risk of re-occurrences is identical in antidepressant and placebo arms (66).

Some authors suggest that long-term antidepressant use may increase the vulnerability for (recurrent) depression due to neurochemical sensitization (58, 59). In support of this thesis, a recent meta-analysis revealed that the relapse risk after antidepressant discontinuation correlates positively with the duration of preceding acute therapy (57). That is, the longer antidepressant treatment, the higher the relapse risk after discontinuation. Moreover, several meta-analyses showed that psychotherapy reduces the long-term relapse risk significantly more than pharmacotherapy, despite both therapies being equally effective during acute therapy (67, 68). A systematic review of observational long-term studies found that maintenance therapy conveys no clear benefits: antidepressant users had no better long-term outcome than non-users (69). In an observational study of patients with remitted recurrent depression, maintenance therapy related to a higher recurrence rate than discontinuation (70). Other observational studies likewise suggest that antidepressant use may worsen the long-term outcome (71, 72), but causality is uncertain due to confounding by indication. A few highly cited epidemiologic studies reported that long-term antidepressant medication relates to lower relapse rates, but these have been shown to be methodologically flawed: when properly analyzed, maintenance therapy is not associated with lower relapse rates (73). Finally, according to the re-analysis of STAR*D (54), only 5.8% of all patients who entered continuation therapy were still in remission after 12-month follow-up. A total of 37.4% of remitted patients, and altogether 64.4% of improved patients had a relapse within the first 12 months of continuation therapy.

## Severe Harm

In children, adolescents and young adults up to 25 years, several meta-analyses of short-term RCT have confirmed that antidepressants, relative to placebo, significantly increase suicide risk (74–77). Moreover, there is now increasing evidence from several RCT and a few well-controlled observational studies that antidepressants may increase suicide risk in adults of any age. On the basis of Bayes’ statistics, Aursnes et al. (78) calculated that paroxetine, compared to placebo, may cause suicide with a certainty of 98–99%. Baldessarini et al. (79) meta-analyzed long-term RCT for adult MD and found a markedly increased rate of completed and attempted suicides in antidepressant arms compared to placebo. With respect to suicidality, i.e., suicide attempts, self-harm, and suicidal ideation combined, a meta-analysis found that antidepressants convey a 2.5 times increased risk (80). Another meta-analysis found that paroxetine increases suicidality in adults aged 18 years and older by a factor 2.6 (51). Gunnell et al. (81) found weak meta-analytic evidence for a slightly increased risk of self-harm in antidepressant users, but not with respect to suicides or suicidal ideation. The recent meta-analysis by Sharma et al. (75) did not find increased suicidality in adult antidepressant users relative to placebo. In contrast to that, Stone et al. (76) meta-analyzed data from pharmaceutical companies submitted to the FDA and found that suicidality was slightly increased in *placebo groups* for adults aged 25–64 years (relative risk: 1.3), and it was even markedly increased in adults aged 65 years and older (relative risk: 2.7). These findings suggest that antidepressants may protect against suicide in middle-aged and older adults, which conflicts with the findings of all other meta-analyses detailed above. However, the work by Stone et al. (76) was criticized, because many cases of suicidality were evidently missing in antidepressant treatment arms (50). In accordance, many authors concluded that industry-funded trials are unreliable, as they willingly underreport cases of suicidality in antidepressant arms (80, 81), for instance by coding suicide attempts as “emotional lability” (75).

Just as the apparent efficacy of antidepressants is overestimated due to publication and reporting biases (15, 23), the pharmaceutical industry conceals harms by underreporting serious adverse events (14, 52, 82). Because RCT typically excludes severely impaired persons with suicidal ideation (35, 36), they run the risk to overlook a pernicious risk. Therefore, industry-funded research systematically underestimates the harm caused by their marketed drugs (14). Although scientifically less stringent (due to confounding by indication), I therefore need to consider well-controlled observational studies conducted by researchers without COI. An advantage of observational studies is that they can encompass much longer time frames than the short-term RCT typically lasting only 6–8 weeks. Due to much larger samples, observation studies allow for detecting harms occurring at low absolute frequency (34). A large naturalistic study with close to 240,000 persons with MD aged between 20 and 64 years found that antidepressant users committed 2.6 times more often suicide than non-users (83). Another well-controlled observational study based on a national register of 5,866 suicides showed that antidepressant use increases the odds of committing suicide 2.7 times in women and 4.3 times in men, with a clear increase in risk with higher age (84). Finally, several ecologic studies supposedly show a negative correlation between national antidepressant sales/prescriptions and suicide rates (suggesting that antidepressants prevent suicide), but these studies are substantially flawed (85) and some were clearly disconfirmed (3, 5).

Naturalistic studies with high power have further shown that antidepressant use prospectively relates to all-cause mortality. For instance, in a study with over 60,000 patients with MD aged 65 years and older, it was shown that over a mean follow-up of 5.0 years, prescription of tricyclics increase the relative mortality rate by 16%, SSRI by 54%, and other antidepressants (mostly SNRI) by 66% (86). In another prospective observational study including over 136,000 postmenopausal women, it was shown that SSRI increase the relative mortality rate by 32% and tricyclics by 67% (87). Finally, a recent meta-analysis showed that antidepressants increase the all-cause mortality risk by 33% in the general population with no difference between SSRI and tricyclics (88). Due to thorough multivariate statistical modeling, it is unlikely that confounding by indication may account for all these effects. Moreover, in psychiatric outpatients, antidepressant use is higher in persons with low distress (89), whereas in primary care, most antidepressants are prescribed to persons without a psychiatric diagnosis (90). Hence, it is possible that confounding by indication may even underestimate the true harm attributable to antidepressant use. Therefore, a reasonable conclusion is that antidepressants disrupt adaptive bodily processes such as digestion, immune function, tissue repair, metabolism, etc., which may lead to premature death (91, 92).

## Conclusion

The results of this mini-review suggest that the efficacy of antidepressants is systematically overestimated, while potential harms are underreported and neglected. Despite these alarming findings, thought leaders within mainstream psychiatry and official treatment guidelines strongly recommend antidepressant use for acute and long-term therapy. However, mainstream psychiatry is closely tied to the pharmaceutical industry, and most leading psychiatric experts receive substantial amounts of financial support from the industry (9, 11, 21). Thus, it is crucial to reiterate that industry-funded trials and research conducted by authors with financial COI is systematically biased toward the pharmaceutical industry’s vested interests (13, 14, 20). The strong alliance between scientific psychiatry and the pharmaceutical industry is problematic and should be subjected to close scrutiny (9–11, 24). Moreover, there is an ongoing debate, whether antidepressant therapy is more efficacious in severe, rather endogenous MD compared to milder, reactive MD (27). Some meta-analytic evidence suggests that this is indeed the case (93), but other failed to replicate this finding (40). Finally, the evidence reviewed in this article relied mostly on RCT, which are also subject to bias (34, 94). Theoretically, these biases could result in an underestimation of antidepressant efficacy, but evidence from real-world effectiveness trials strongly argues otherwise, since real-world patients are more impaired and respond significantly worse to antidepressant therapy (36, 37). Finally, it has been argued that the efficacy of antidepressants is underestimated in more recent trials due to marked increases in placebo responses over time. However, that claim is false. A recent meta-analysis has clearly shown that the placebo response rate has been stable for almost 30 years now (95). As concerns treatment recommendations, my reading of the literature is that some patients may benefit from acute pharmacotherapy, but on average clinical benefits are debatable and should be weighed against adverse side effects (38, 39, 51). Continuation and maintenance therapy is not recommended due to an apparent lack of clear clinical benefits (54, 55, 96), coupled with a possibly increased vulnerability to chronic depression (58, 97), increased suicide risk (79, 83), and, in particular in older adults, higher all-cause mortality (86, 88, 98).

## Author Contributions

MH conducted the literature review and wrote the entire manuscript.

## Conflict of Interest Statement

The author declares that the research was conducted in the absence of any commercial or financial relationships that could be construed as a potential conflict of interest.
